# The Health Economic Impact of Musculoskeletal Physiotherapy Delivered by Telehealth: A Systematic Review

**DOI:** 10.5195/ijt.2023.6524

**Published:** 2022-12-13

**Authors:** Darryn Marks, Sarah Kitcher, Elodie Attrazic, Wayne Hing, Michelle Cottrell

**Affiliations:** 1 Bond University, School of Physiotherapy, Gold Coast, Australia; 2 Gold Coast University Hospital, Gold Coast, Australia; 3 Physiotherapy Department, Royal Women's and Brisbane Hospital, Australia

**Keywords:** Cost, Efficiency, Musculoskeletal, Physiotherapy, Telerehabilitation

## Abstract

**Introduction:**

While the efficacy of telehealth in musculoskeletal physiotherapy has been supported, its cost effectiveness has not been established. Therefore, the objective of this review was to ascertain the health economic impact of outpatient musculoskeletal physiotherapy delivered by telehealth and describe methodology utilized to date.

**Methods:**

Electronic searching of PubMed, CINHAL, PEDro, and Web of Science databases was undertaken alongside handsearching for publications comprising: population: adults with musculoskeletal disorders managed in any type of outpatient ambulatory setting; intervention: physiotherapy delivered by telehealth comparison: traditional in-person physiotherapy; and, outcomes: economic analyses reporting costs and consequences. Appraisal was undertaken with the Downs and Black Questionnaire and the Consolidated Health Economic Evaluation Reporting Standards Checklist.

**Results:**

Eleven studies of mixed methodological quality were included. Most were conducted in the public sector, from the economic perspective of the health service funder. Telehealth consistently produced health outcomes akin to in-person care. In all but one, telehealth was less costly, with savings achieved by reducing in-person consultations and travel costs.

**Conclusion:**

Telehealth is as effective and cheaper than in-person physiotherapy for musculoskeletal disorders in public hospital outpatients. Further health economic research is needed to clarify the economic impact of telehealth upon non-government providers of musculoskeletal physiotherapy.

Musculoskeletal disorders (MSD) represent the leading cause of disability across the globe and are expected to increase in prevalence as the population ages (WHO, 2021), thereby placing growing pressure on already challenged health systems (Curtis et al., 2010). Some propose that telehealth could ease this burden by improving the efficiency of care (De Guzman et al., 2021) but the health economic impact of telehealth for MSD remains unclear. Multiple reviews have reported the economic impact of telehealth to be either under-researched or unclear (de la Torre-Díez et al., 2015; Mistry, 2012; Snoswell et al., 2020; Tsou et al., 2020) and no prior reviews have specifically investigated the economics of telehealth in musculoskeletal physiotherapy.

Telehealth encompasses the breadth of information and communication technology aids to the delivery of health care. It can occur synchronously (in real-time) between patient and clinician (telephone, videoconferencing), or asynchronously (smart-device applications, web-based platforms). It can be used in conjunction with traditional in-person care (hybrid care model) or for entire care episodes. For many musculoskeletal physiotherapists, the SARS-CoV-2 (COVID-19) pandemic necessitated the rapid implementation of telehealth, with a wide variety of platforms used (Cottrell & Russell, 2020; Malliaras et al., 2021). In this period, physiotherapists in mixed settings reported positive experiences with videoconferencing (Bennell et al., 2021). Yet a sample of predominantly musculoskeletal physiotherapists in private practice, were less positive about the value of telehealth (Malliaras et al., 2021). Outcome literature has shown telehealth in musculoskeletal physiotherapy to be clinically effective in the management of hip and knee arthroplasty (Agostini et al., 2015; Castrodad et al., 2019), post shoulder surgery (Pastora-Bernal, Martín-Valero, Barón-López, et al., 2018), osteoarthritis (OA) and chronic low back pain (Du et al., 2020; Pietrzak et al., 2013). Yet others are more cautious, finding videoconferencing to be as effective as traditional practise for many, but not all, musculoskeletal assessments (Mani et al., 2017). A recent systematic review of videoconferencing specific to musculoskeletal physiotherapy reported positive impacts on health outcomes and satisfaction but a lack of evidence on economic impact (Grona et al., 2018).

It is important to note that telehealth implementation should be tailored to the specific requirements of a health service (Cottrell & Russell, 2020) and as musculoskeletal physiotherapy spans a wide range of settings with different funding structures, extrapolation of economic data across such varied settings may require caution. Australian workforce data indicates that 72% of physiotherapists work in the private sector, the majority (53%) with musculoskeletal clientele and furthermore, small group or solo private practices are the workplace of 46% (Australian Government Department of Health, 2019). This Australian example serves to illustrate risks associated with over-generalization of economic data, as conclusions about the economic impact of telehealth drawn from research conducted in large public hospitals, may lack applicability to almost half Australian practitioners, who work in small or solo private practices. Therefore, illumination of the health economic methodology and context underpinning conclusions about the value of telehealth, is needed so that applicability to different work settings can be judged. No prior reviews have undertaken synthesis of this information with respect to telehealth in musculoskeletal physiotherapy. This review therefore aims firstly to synthesize the evidence pertaining to the health economic impact of telehealth in the outpatient musculoskeletal physiotherapy setting, and secondly to describe the health economic methodology used to date.

## Methods

This systematic review followed the Preferred Reporting Items for Systematic Reviews Guidelines (Page et al., 2021). The protocol was registered with the PROSPERO international prospective register of systematic reviews; PROSPERO 2020 CRD42020198720 Available from: https://www.crd.york.ac.uk/prospero/display_record.php?ID=CRD42020198720

### Eligibility Criteria

Included study designs were those published in the English language that compared a telehealth intervention with usual physiotherapy care, regardless of study type.

Population: Included were adult patients with musculoskeletal disorders managed in any type of outpatient/ambulatory (non-inpatient) setting. Non-musculoskeletal disorders, including systemic inflammatory diseases, as well as musculoskeletal conditions that were the direct result of another medical co-morbidity (e.g., shoulder subluxation post-stroke), were excluded.

Intervention: Included were studies in which physiotherapy was delivered by telehealth in isolation (pure telehealth intervention) or in part (telehealth combined with usual care), using any form of synchronous or asynchronous information communication technology (ICT). Interventions delivered by a health profession other than physiotherapy were excluded.

Comparison: Studies in which physiotherapy care was provided in-person were included, while those that did not involve conventional physiotherapy care or that compared two different types of telehealth interventions were excluded.

Outcomes: Studies that included outcomes which assessed full health economic analyses reporting costs and consequences were included. Studies without presentation of costs and consequences were excluded.

### Data Sources and Search Strategy

Four electronic databases were searched (PubMed, CINHAL, PEDro and Web of Science) from inception until November 2021. The search included terms based around key terms of physiotherapy, telehealth, musculoskeletal disorders, and economics. An example of the full search strategy is provided (Appendix A). Handsearching of reference lists from studies and relevant literatures reviews was also undertaken.

### Study Selection

**Figure 1:**
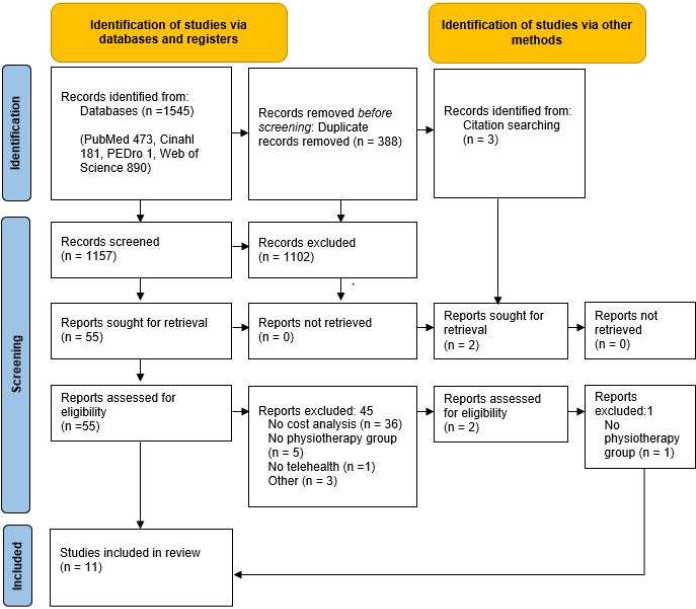
Preferred Reporting Items for Systematic Reviews and Meta-analyses (PRISMA) 2020 Flow Diagram (Page et al., 2021)

*Note.* From the PRISMA 2020 statement: An updated guideline for reporting systematic reviews. For more information, visit: http://www.prisma-statement.org/

Study selection was undertaken independently by two reviewers (DM, MC) with disagreement resolved by involvement of a third reviewer as required. Studies were first screened by title and abstract, and full text of remaining studies were retrieved and assessed against the selection criteria. Search results were uploaded onto a reference management software (Endnote, Version 20 Clarivate). The PRISMA diagram (Figure 1) details the results at each step within this process.

### Data Extraction

Data extraction was undertaken independently by two reviewers (DM, SK) using a data template and processes based on those recommended by the Joanna Briggs Institute (Aromataris, 2020) and included study details such as type, location and setting, condition being treated, intervention, methodological features, health and economic outcomes and results. Input from a third reviewer was sought if disagreements were not resolved with discussion.

### Critical Appraisal/Assessment of Methodological Quality

Included studies were assessed for methodological quality using both a modified version of the Downs and Black instrument (Appendix B) and the Consolidated Health Economic Evaluation Reporting Standards (CHEERS) checklist (Appendix C). The Downs and Black instrument has been validated in non-randomised and randomised trials (Downs & Black, 1998) and has good validity for studies of varied design (Deeks et al., 2003; Hootman et al., 2011). It was initially described with 27 questions and a score out of 32, as one question is scored out of 2 and another out of 5. We applied a widely adopted modification (Morton et al., 2014; O'Connor et al., 2015) in which the final question in relation to power, was condensed from a score out of 5, to a score of 0 or 1. This results in a final score out of 28. The CHEERS checklist (Husereau et al., 2013) is a 24-item quality assessment tool which can be used to assess the methodological quality of health economic evaluations. Each included study was independently assessed by two independent reviewers (SK, DM). Differences were resolved by discussion and consensus with a third researcher (MC).

### Data Synthesis and Analysis

A descriptive synthesis was conducted. Due to the heterogeneity of methodologies, perspectives, and health economic outcomes within the included studies, a meta-analysis was not undertaken.

Studies were assigned to the following health economic categories according to their methodology: *Cost consequence analysis (CCA)* which examines costs and consequences without isolation or aggregation of consequences into a single measure; *Cost effectiveness analysis (CEA)* which usually relate the costs of two alternative treatments to the same consequence presented as a cost per increment of effectiveness; *Cost minimization analysis (CMA)* in which the consequences of each intervention are known to be equal and thus only a cost comparison is required; *Cost utility analysis (CUA)* which is a special case of the CEA in which the consequences of the alternative treatments are compared with the metric of quality adjusted life years (QALYS) derived from health utility scores (Drummond, 2005; Husereau et al., 2013; Snoswell et al., 2020). In cases where the perspective of the health economic analysis was either not stated or unclear, a perspective was determined from the costing/monetization methodology provided within the study.

## Results

### General Description of Included Studies

Eleven studies of mixed methodological quality met the inclusion criteria, as detailed in Figure 1. The details of included studies are summarized in Tables 1 and 2. Six studies presented health economic analyses accompanying an RCT (Bettger et al., 2020; Fatoye et al., 2020; Hollinghurst et al., 2013; Kloek et al., 2018; Pastora-Bernal, Martín-Valero, & Barón-López, 2018; Tousignant et al., 2015) and of these, three published cost analyses separately (Hollinghurst et al., 2013; Kloek et al., 2018; Pastora-Bernal, Martín-Valero, & Barón-López, 2018). The remaining studies consisted of health economic analyses accompanying a prospective audit (Beard et al., 2017), a retrospective audit (Cottrell et al., 2019), a retrospective cohort (Zachwieja et al., 2020), a prospective cohort (Mallett et al., 2014) and a matched cohort study (Horton et al., 2021). A variety of health economic analyses, conditions and settings were presented, which precluded meta-analysis. A variety of musculoskeletal conditions appeared; five presented post-operative patients (knee arthroplasty (Bettger et al., 2020; Tousignant et al., 2015; Zachwieja et al., 2020), hip arthroscopy (Horton et al., 2021), shoulder subacromial decompression (Pastora-Bernal, Martín-Valero, & Barón-López, 2018), two back pain (Beard et al., 2017; Fatoye et al., 2020), three mixed MSD (Cottrell et al., 2019; Hollinghurst et al., 2013; Mallett et al., 2014) and one hip/knee osteoarthritis (Kloek et al., 2018).

**Table 1:** Research Methodology Including Telehealth Interventions, Health-related Results and Quality Appraisal of Included Studies

Study	Design	Condition	Setting	Telehealth Intervention^*^	n	Health and service outcome measures	Results	Downs & Black/28
Beard (Beard et al., 2017)	Prospective audit	Back pain	Australia Public hospital	VC with patient and local physiotherapist in remote setting.	41	Wait times, attendance, safety, discharge, care rates	Descriptive statistics showing similar findings both groups	19
Bettger (Bettger et aL, 2020)	RCT	Post knee arthroplasty	USA Private Clinics	Online avatar to guide exercise, 3D tracking to monitor activity performance, VC. Able to receive in-person visits if deemed required.	306	KOOS, PROMIS, ROM, Gait Speed, readmission.	Non-inferiority declared for all measures	23
Cottrell (Cottrell et aL, 2019)	Retrospective audit	Mixed	Australia Public hospitals	VC with patient and local physiotherapist in remote setting.	44	Discharge/referral rates, safety	Descriptive statistics showing similar findings both groups	19
Fatoye (Fatoye et aL, 2020)	RCT	Back pain	Nigeria Public hospital	Physiotherapy (McKenzie) by mobile phone application. Feedback and progress monitored with SMS and phone calls for 8 weeks	56	Oswestry Disability Index	No significant difference between groups.	18
Hollinghurst (Hollinghurst et aL, 2013)	RCT	Mixed	UK Public hospitals	PhysioDirect initial consultation (assessment and advice) by telephone followed by in-person care as required.	2249	SF36, EQ5D3L, Measure Yourself, Global Improvement Score, Satisfaction, Waiting time	No significant differences on health outcomes. More and longer consultations and waits in usual care, lower satisfaction in PhysioDirect	21
Horton (Horton et aL, 2021)	Matched cohort	Post Hip arthroscopy	USA Private Hospital	Initial in-person then telehealth 3 months exercises via VC portal.	51	iHOT	No significant difference between groups	15
Kloek (Kloek et aL, 2018)	RCT	Hip/knee OA	Netherlands Public hospital	5 in-person sessions and thereafter a web-application of exercise and education for 12 weeks total.	207	HOOS, KOOS, Activity via accelerometers, EQ5D3L	No significant health difference between groups and fewer appointments for telehealth group.	23
Mallett (Mallett et al., 2014)	Prospective Cohort	Mixed	UK Public hospital	PhysioDirect initial consultation (assessment and advice) by telephone followed by in-person care as required.	194	EQ5D5L, attendance, appointments, satisfaction	No significant difference in health. Significantly less non-attendance, appointments, and greater satisfaction in telehealth group	14
Pastora-Bernal (Pastora-Bernal, Martin-Valero, & Baron-Lopez, 2018)	RCT	Post shoulder arthroscopy	Spain Public hospital	VC and exercise videos 5 days a week for 12 weeks via email, also educational material.	18	Constant Murley, WOMAC	No significant difference between groups. Non-inferiority reported without statistical support)	22
Tousiqnant (Tousignant et al., 2015)	RCT	Post knee arthroscopy	Canada Public hospitals	VC twice a week for 8 weeks	197	WOMAC	No significant difference between groups	23
Zachwieja (Zachwieja et al., 2020)	Retrospective cohort	Post knee arthroplasty	USA Private Hospital	Web-based exercises, emails, videos, and online access to clinicians. Option of in-person visits.	701	KOOS, Veterans RAND Short Form, MUA rate, ROM	No significant difference between groups	19

*Note*. KOOS: Knee injury and Osteoarthritis Outcome Score; PROMIS: Patient-Reported Outcomes Measurement Information System; ROM: Range of motion; RCT: Randomized controlled trial; MUA: Manipulation under anaesthesia; iHOT: International hip outcome tool; WOMAC: Western Ontario and McMaster Universities Osteoarthritis Index; VC: videoconference.

* In each study telehealth was compared with usual in-person physiotherapy care, Tousignant et al via home-visits

**Table 2:** Health Economic Methodology, Results, and Quality Appraisal of Included Studies

Study	Type	Economic perspective/s	Costed items, (costing method)	Health Economic Outcome	Main Reason	CHEERS/24
Beard (Beard et al., 2017)	CCA	Health Service (Funder-Government)	Staff time preparation, delivery, travel. Patient travel. (Standard public sector rates).	Telehealth cheaper (AU S11187) with similar health service outcomes to usual care (AU $14452), over 5 months of service.	Reduced staff travel costs	13
Bettger (Bettger et al., 2020)	CCA	Health Service (Funder-Government)	Tally of care, staff time for telehealth (Medicare fee-for-service, wage rates).	Telehealth significantly cheaper per episode (US $1050) and noninferior to usual care (US $2805).	Fewer appointments	20
Cottrell (Cottrell et al., 2019)	CCA	Health Service (Provider - Public)	Staff time in training, preparation and delivery, staff travel. (Standard public sector rates).	Telehealth cheaper (AU $11930) with similar health service outcomes compared to usual care (AU S13699) over 9 weeks.	Reduced staff travel costs	19
Fatoye (Fatoye et al., 2020)	CUA	Patient	Consultation tally. Patient expenses including travel, phone use, refreshments. (Fixed consultation cost. Patient reported costs).	Telehealth cheaper (US 561.70), with no significant difference in health outcomes compared with usual care (US $106) over 8 weeks. ICER: Telehealth Dominant (0.001 higher QALY in telehealth group)	Reduced patient travel costs	16
Hollinghurst (Hollinghurst et al., 2013)	CUA	Health Service (Funder-Government)	Staff time, overheads, prescriptions, other NHS services. Patient reported phone, medications equipment, travel, loss of earnings and productivity, absence for care, equipment, domestic help, private therapy. (Standard public sector rates. Patient reported costs).	Physiotherapy alone: telehealth (£74.01) costlier than usual (£69.73) per 6-month episode with no significant difference in health outcomes. CUA (Health service perspective (total NHS costs)): Telehealth more expensive (£198.98) and more effective (QALY 0.332), compared with usual care (£179.68 and QALY 0.325), ICER: £2889. Aspects of patient and societal perspectives collected but not calculated.	Reduced travel costs	20
Horton (Horton et al., 2021)	CCA	Health Service (Funder)	Tally of services provided. (Hospital billed charges).	Telehealth (US $1015.67) significantly cheaper than usual care with same PTs (US $1555.62) and different PTs (US $1896.38) over 3 months, with no significant difference in health outcomes.	Fewer appointments	14
Kloek (Kloek et al., 2018)	CUA	Societal and Health Service (Funder-Government)	Tally of healthcare, sports, informal care, patient-reported absenteeism/presenteeism. (Standard public sector service payments).	Societal: costs per participant not significantly different for telehealth (Euro 6348) compared to usual care (Euro 7718), no significant difference in health outcomes. CUA Societal: TH dominant TH 529E cheaper and 0.01 QALY better, ICER-52900 Health Sector: TH also dominant, ICER-79200.	Fewer appointments	23
Mallett (Mallett et al., 2014)	CMA	Health Service (Funder-Government)	Tally of care provision. (Standard public sector service payments).	Telehealth £36.42 cheaper per episode (individual group data not provided). CMA conducted, despite inconclusive efficacy of this telehealth model.	Fewer appointments	12
Pastora-Bemal (Pastora-	CCA	Health Service (Funder-Government)	Staff time in training, preparation, and delivery. (Standard public sector rates.	Telehealth significantly cheaper (Euro 236.97) than usual care (Euro 304.42) per participant over 12 weeks, with no significant health difference between groups	Fewer appointments	16
Bernal, Martin-Valero, & Baron-Lopez, 2018)			Fixed telehealth license and technical rates).			
Tousignant (Tousignant et al., 2015)	CCA	Health Service (Funder-Government)	Staff time in training, preparation, and delivery. (Standard public sector rates).	Telehealth (CAN $1224) significantly cheaper than in-person home visits (CAN $1487) and different PTs (US $1896.38) over 8 weeks, with no significant difference in health outcomes. Staff travel <30km no significant difference but >30km telehealth significantly cheaper.	Reduced staff travel costs	21
Zachwieja (Zachwieja et al., 2020)	CCA	Health Service (Funder-Government)	Tally of care provision. (Fixed fee per case for telehealth. Per episode cost collected from commercial payers).	Telehealth (US $100) significantly cheaper than totally in-person care (US $1444) over 6 months, with no significant difference in health outcomes. Other groups with combinations of in-person and online care were not significantly different.	Fewer appointments	19

*Note*. CCA: cost consequences analysis; CUA: cost utility analysis; CMA: cost minimization analysis; TH: telehealth; ICER: incremental cost effectiveness ratio; PT: Physical Therapist

### Quality and Risk of Bias

Modified Downs and Black scores ranged from 14 to 23 out of a possible 28. CHEERS scores ranged from 12 to 23 out of a possible 24. These scores are presented alongside study details in Tables 1 and 2, with full scoring details provided in the appendices. General quality themes included low sample sizes, a lack of clarity around equivalence of health outcomes and non-inferiority in some studies. There was also a lack of detail and clarity surrounding costing, monetization processes and perspectives presented in some studies.

### Settings

Eight studies were conducted from the public hospital outpatient settings (Beard et al., 2017; Cottrell et al., 2019; Fatoye et al., 2020; Hollinghurst et al., 2013; Kloek et al., 2018; Mallett et al., 2014; Pastora-Bernal, Martín-Valero, & Barón-López, 2018; Tousignant et al., 2015). Three studies were conducted from private (non-public) healthcare settings, of which two were private hospital outpatients (Horton et al., 2021; Zachwieja et al., 2020) and one in private medical centres and independent private clinics although cost analysis in this study was based on public rates (Bettger et al., 2020). In all but one study the usual in-person physiotherapy care arm of the study was based within the settings listed above. One study exclusively provided home visits for the in-person care (Tousignant et al., 2015). Telehealth interventions were delivered to patients within their own home, except for two studies in which patients attended a remote facility for videoconferencing (Beard et al., 2017; Cottrell et al., 2019).

### Telehealth Interventions

All studies provided synchronous telehealth interventions. In eight studies the intervention was mixed with both telehealth and in-person care (Beard et al., 2017; Bettger et al., 2020; Cottrell et al., 2019; Fatoye et al., 2020; Hollinghurst et al., 2013; Horton et al., 2021; Kloek et al., 2018; Mallett et al., 2014), two provided a pure telehealth intervention (Pastora-Bernal, Martín-Valero, & Barón-López, 2018; Tousignant et al., 2015) and one provided both pure and mixed interventions (Zachwieja et al., 2020). While the majority of mixed intervention groups contained substantial telehealth elements, in two studies only the initial assessment consultation was by telehealth with subsequent care in person (Hollinghurst et al., 2013; Mallett et al., 2014). Videoconferencing was the most frequent telehealth delivery medium, being used in five studies (Beard et al., 2017; Cottrell et al., 2019; Horton et al., 2021; Pastora-Bernal, Martín-Valero, & Barón-López, 2018; Tousignant et al., 2015). Three studies provided an integrated online web-based platform with a combination of information, email and communication such as videoconference (Bettger et al., 2020; Kloek et al., 2018; Zachwieja et al., 2020). Three studies used telephony, of which two were studies in which that initial assessment consultation was conducted by phone and thereafter care was in-person (Hollinghurst et al., 2013; Mallett et al., 2014) and one study used mobile phone applications and communication (Fatoye et al., 2020).

## Health Outcomes

### Summary

Telehealth has consistently produced health outcomes similar to usual care. A wide variety of health outcome measures and health service-related measures such as waiting times, appointments, and referrals, were reported across included studies. Details of the outcome measures used in each study are provided in Table 1. In all 11 included studies, the health and/or service outcomes of telehealth intervention were either non-inferior (Bettger et al., 2020), not significantly different (Fatoye et al., 2020; Hollinghurst et al., 2013; Horton et al., 2021; Kloek et al., 2018; Mallett et al., 2014; Pastora-Bernal, Martín-Valero, & Barón-López, 2018; Tousignant et al., 2015; Zachwieja et al., 2020) or descriptively similar (Beard et al., 2017; Cottrell et al., 2019) to that of usual in-person physiotherapy care. No studies found usual in-person care to be superior to telehealth.

## Health Economic Methodologies

### Summary

Few studies integrated health consequences and costs into cost-effectiveness or cost-utility analyses. The health service perspective is well represented, specifically public hospitals funded by the government. Other perspectives (private hospitals, provider, patient, societal) are sparse, and the impact of telehealth from the perspective of private providers is absent.

### Economic Analyses

Health economic methodologies used within the included studies are presented in Table 2. Cost consequences analyses were the most common. This type of analysis investigates costs and consequences without aggregating to single measures such as quality adjusted life years (Husereau et al., 2013). These appeared in seven studies (Beard et al., 2017; Bettger et al., 2020; Cottrell et al., 2019; Horton et al., 2021; Pastora-Bernal, Martín-Valero, & Barón-López, 2018; Tousignant et al., 2015; Zachwieja et al., 2020). There were three cost utility analyses which converted health utility scores to QALYs and calculated an incremental cost effectiveness ratio (Fatoye et al., 2020; Hollinghurst et al., 2013; Kloek et al., 2018). In one of these, a quality of life outcome measure was not completed by participants and instead health utility scores used calculate QALYs were estimated by converting Oswestry Disability Index to short-form six dimension scores using a previously published formula (Fatoye et al., 2020). The extent to which this process assessed normative population data is unclear. Outside of these three CUAs, no studies presented cost and consequences as a cost per increment of effectiveness, thus no studies met the criteria of cost effectiveness analyses. One study conducted a cost minimization analysis, in which the health consequences of the interventions being compared are known to be equivalent (Husereau et al., 2013). However, in this study (Mallett et al., 2014) the evidence presented to support equivalence of efficacy was questionable.

### Perspectives

Table 2 details and Table 3 summarizes the perspectives presented in each study. Ten of the 11 included studies presented a health service perspective (one study did this in addition to a primary societal analysis (Kloek et al., 2018)). Of these, nine were from the funder's perspective, with all but one set in the public sector and thus the government was the funder of services (Beard et al., 2017; Bettger et al., 2020; Hollinghurst et al., 2013; Kloek et al., 2018; Mallett et al., 2014; Pastora-Bernal, Martín-Valero, & Barón-López, 2018; Tousignant et al., 2015; Zachwieja et al., 2020). One presented a health service funder perspective, in which parties other than the government were funders (Horton et al., 2021). One study presented a health service perspective with cost analysis from the perspective of a public sector provider, in which provider costs and revenue from the government was presented (Cottrell et al., 2019). There was one full societal perspective (Kloek et al., 2018) and one patient perspective (Fatoye et al., 2020), and no studies from the perspective of a private provider.

**Table 3:** Overview of Health Economic Perspective, Results, and Quality

Perspective of analysis	Telehealth cheaper		Telehealth more expensive	
	Study	CHEERS	Study	CHEERS
Health Service - Funder (public)	Beard (Beard et al., 2017)	12	Hollinghurst (Hollinghurst et al., 2013)^*^^^^	20
	Bettger (Bettger et al., 2020)			
	Kloek (Kloeketal., 2018)^*^^^^	19		
	Mallett (Mallett et al., 2014)^*^	11		
	Pastora-Bemal (Pastora-Bemal, Martín-Valero, & Barón-López,2018)	16		
	Tousignant (Tousignant et al., 2015)^#^	21		
	Zachwieja (Zachwieja et al., 2020)	19		
Health Service - Funder (private)	Horton (Horton etal., 2021)	14	Nil			
Health Service - Provider (public)	Cottrell (Cottrell et al., 2019)^*^	19				
Societal	Kloek (Kloek et al., 2018)^*^^^^	21				
Patient	Fatoye (Fatoye et al., 2020)^^^	16				

* Findings in favour of telehealth intervention but significance of cost comparison not reported or not achieved

^ CUA presented supporting cost-effectiveness of telehealth

# Comparator home visits <30km travel distance not significantly cheaper

## Health Economic Impact of Telehealth

### Summary

There is consistent evidence that telehealth provides similar health outcomes at lower cost than usual in-person care for a wide variety of musculoskeletal conditions from the perspective of the health service funder (government) in the public setting. While there is limited evidence from other perspectives, there is an indication that telehealth is also more cost efficient for public providers, private hospital funders and society. There is insufficient evidence to draw conclusions about cost-efficiency for private non-hospital providers.

The health economic results of included studies are presented in Table 2 and summarized by perspective in Table 3.

### Cost Consequences Analyses (CCA)

Seven CCAs with CHEERS scores ranging from 13 to 21, spanning a wide range of pre and post operative musculoskeletal conditions, reported that telehealth delivers health outcomes akin to usual care and at less expense than in-person care from the perspective of the health service. Five were from the perspective of the government as the funder of the public hospital services (Beard et al., 2017; Bettger et al., 2020; Pastora-Bernal, Martín-Valero, & Barón-López, 2018; Tousignant et al., 2015; Zachwieja et al., 2020), one from the perspective of a public health provider of services (Cottrell et al., 2019) and one from the perspective of the funder of private hospital care (Horton et al., 2021). One study used a comparator of home visits rather than a model of care in which patients attended a clinic (as was the comparator in other studies), finding that cost-effectiveness of telehealth was dependent upon a travel distance >30km but not significantly different when professionals travelled <30km (Tousignant et al., 2015).

### Cost Utility Analyses (CUA)

Three studies spanning mixed musculoskeletal presentations, hip, knee OA and back pain, with CHEERS scores from 16–23 presented a CUA and incremental cost effectiveness ratio (ICER). Two of these showed a dominant result in favor of telehealth for treating back pain from the patient perspective (Fatoye et al., 2020) and hip/knee OA from societal perspective and health service perspectives (Kloek et al., 2018), indicating that telehealth was both cheaper and more effective than usual care. The latter also noted that costs were not significantly different between groups. The third (Hollinghurst et al., 2013) found the PhysioDirect telehealth model to be both more expensive and marginally more effective than usual care. All of these studies reported no difference in effectiveness between telehealth and usual care, according to the primary health outcomes used in each study. In each case, QALY findings marginally in favor of telehealth (0.001 (Fatoye et al., 2020), 0.007 (Hollinghurst et al., 2013) and 0.01 (Kloek et al., 2018)) influenced the ICER in favor of telehealth.

### Cost Minimization Analysis (CMA)

One CMA also conducted in a public hospital from the funder perspective and with a CHEERS score of 12, also found telehealth to be cheaper when compared to usual care (Mallett et al., 2014).

### Drivers of Cost Savings with Telehealth

Across included studies two main features of telehealth were consistently apparent as the key drivers of cost efficiency: the need for fewer (more costly) in-person appointments in six studies (Bettger et al., 2020; Hollinghurst et al., 2013; Kloek et al., 2018; Mallett et al., 2014; Pastora-Bernal, Martín-Valero, & Barón-López, 2018; Zachwieja et al., 2020), and reduced travel-related costs for staff and/or patients in five studies (Beard et al., 2017; Cottrell et al., 2019; Fatoye et al., 2020; Hollinghurst et al., 2013; Tousignant et al., 2015).

## Discussion

This is the first systematic review to specifically investigate the health economic impact of telehealth in outpatient musculoskeletal physiotherapy and synthesize the methodologies used to arrive at efficiency judgements. It shows that the quality of health care is maintained, and care is cheaper, when traditional physiotherapy is replaced or augmented by telehealth for a variety of musculoskeletal conditions managed in a public hospital setting and when considered from the perspective of the government that funds such services. There is a lack of research in other settings and from other perspectives, but a small number of studies suggest telehealth for outpatient musculoskeletal care may also be efficient for public providers, patients, and society. A prior musculoskeletal physiotherapy review of videoconferencing (Grona et al., 2018), contained only two cost studies and reported inconclusive economic findings. Our wider telehealth and comparator definitions and increased focus on health economics led to the inclusion of 11 papers. While this did create greater heterogeneity of care (two studies involved only initial consultation by telehealth (Hollinghurst et al., 2013; Mallett et al., 2014) and one a study conducted usual care by home visit (Tousignant et al., 2015)) a more comprehensive synthesis of the economic literature in this field has been achieved.

As previously reported (Snoswell et al., 2020), telehealth reduced travel costs for staff and patients, and reduced in-person appointments in telehealth interventions in all included studies. Only three studies included incremental cost effectiveness ratios (Fatoye et al., 2020; Hollinghurst et al., 2013; Kloek et al., 2018) and all should be interpreted with caution due to the influence of marginal QALY results in favor of telehealth. Other authors have observed similar marginal QALY improvements with telehealth and interpreted these as incidental findings below what is clinically meaningful (Snoswell et al., 2020). This highlights the need for further robust health economic research in this field with higher sample sizes and greater clarity on the economic perspective being taken so that results can be clearly interpreted, even in the presence of small QALY differentials.

Accurate interpretation and application of economic efficiency assumptions is important. Our findings do not imply that all care should be delivered by telehealth, but rather confirm the potential benefits of integrating telehealth into usual care. This review also highlighted a lack of research into the health economic implications of telehealth for providers of private musculoskeletal physiotherapy. This represents a large group in some countries and often includes small private clinics (Australian Government Department of Health, 2019). With expense and revenue profiles quite different to those of public or private hospitals, extrapolation of the general findings of this review to private sector providers would seem unwise. Understanding the different perspectives of economic analyses is crucial. Cost savings from the health service perspective may be diluted and appear insignificant from a wider societal perspective, amongst larger costs such as work productivity (Kloek et al., 2018). Furthermore, cost savings for funders, may not equate to savings for smaller private providers, particularly if their main revenue source is consultation fees. Further research is needed to establish the potential impact of telehealth upon private providers and explore opportunities to work with the emerging technology in a financially sustainable way.

With regard to clinical efficacy, our results affirm telehealth as an effective option for the management of musculoskeletal disorders in an outpatient setting. This supports prior findings of positive results for intervention studies providing physiotherapy via videoconferencing (Grona et al., 2018). However, with a recent increase in telehealth imposed by COVID-19 pandemic conditions, physiotherapists now more familiar with these platforms have reported both positive experiences across mixed patient populations (Bennell et al., 2021) but also a much more hesitant stance in relation to musculoskeletal disorders specifically; a recent survey of musculoskeletal clinicians, found less than half felt telehealth was as effective as usual care, particularly when care would involve manual assessment and treatment techniques would normally be involved (Malliaras et al., 2021). Further research is indicated, to establish whether subgroups of musculoskeletal patients (or clinicians) do fare better with traditional in-person care. A health economic approach, investigating both efficacy and economic aspects, could provide greater insight into this conundrum.

## Limitations

Several limitations impact this systematic review. The methodological quality of included studies varied widely, while several studies included low sample sizes. Broad telehealth intervention and comparator definitions were chosen to ensure a comprehensive synthesis of the literature but this increased methodological heterogeneity of included studies and precluded meta-analysis. Some studies included costs from multiple perspectives but lacked clarity in their interpretation and analysis of results within their stated health economic perspective. Consequently, we were unable to interpret some of these cost items within the scope of this review. While we acknowledge the challenges of conducting pragmatic clinical research, we have also highlighted the need for future robust health economic research in this field.

## Conclusion

Telehealth delivers similar health outcomes at a lower cost than in-person care for musculoskeletal disorders managed in public hospital outpatient settings and considered from the health service perspective of the government funder. Most savings are made by a reduction in in-person consultations and travel costs. A small number of studies indicate that telehealth may also be efficient from public provider, patient, and societal perspectives but its impact upon private providers of musculoskeletal care is presently unknown. Further robust health economic research is needed to clarify the economic impact of telehealth upon non-government providers of musculoskeletal physiotherapy.

## Appendix A Example search strategy (Pubmed)

(Physiotherapy [title/abstract] OR “Physical therapy” [title/abstract] OR “Physical Therapy Modalities”[Mesh]) AND (Musculoskeletal [title/abstract] OR Muscle [title/abstract] OR Skeletal [title/abstract] OR Knee[title/abstract] OR shoulder[title/abstract] OR back[title/abstract]) AND (Telecare[title/abstract] OR Telemedicine[title/abstract] OR Telemedical [title/abstract] OR Telerehabilitation [title/abstract] OR telehealth[title/abstract] OR telemonitoring [title/abstract] OR Telerehabilitation [Mesh] OR remote [title/abstract] OR teletherapy* [title/abstract] OR telephone [title/abstract] OR video [title/abstract] OR synchronous[title/abstract] OR asynchronous[title/abstract] OR internet[title/abstract]) AND (Economics[Title/Abstract] OR cost*[Title/Abstract] OR (health [Title/Abstract] AND economics[Title/Abstract]) OR financial[Title/Abstract] OR benefits[Title/Abstract] OR outcomes[Title/Abstract] OR fees[Title/Abstract])

## Appendix B Downs and Black Checklist

**Table:**

	Downs and Black Checklist (modified/28)	Beard	Bettger	Cottrel	Fatoye	Hollinghurst	Horton	Kloek	Mallet	Pastora	Tousignant	Zachweija
1	Aim clearly described	1	1	1	1	1	1	1	1	1	1	1
2	Main outcomes clearly described	1	1	1	1	1	1	1	0	1	1	1
3	Patients clearly described	1	1	1	1	1	1	1	0	1	1	1
4	Interventions clearly described	1	1	1	0	1	1	1	1	1	1	1
5	Confounders described (/2)	1	1	1	0	1	0	1	1	1	1	1
6	Main findings clearly described	1	1	1	1	1	1	1	1	0	1	1
7	Estimates of random variability	0	1	1	1	1	1	1	0	1	1	1
8	Adverse events reported	1	1	1	0	0	0	0	0	0	0	0
9	Lost to follow-up described	1	1	0	0	0	0	1	0	1	0	0
10	Probability values reported	0	1	0	1	0	1	0	1	1	1	1
11	Subjects asked representative	1	1	1	1	1	0	1	1	1	1	1
12	Subjects representative	1	1	1	1	1	0	1	1	1	1	1
13	Staff and facilities representative	1	1	1	1	1	1	1	1	1	0	1
14	Subjects blind to the intervention	0	0	0	0	0	0	0	0	0	0	0
15	Blinded measures of main outcomes	0	0	0	1	0	0	1	0	1	1	0
16	Any “Data dredging”	1	1	1	1	1	1	1	1	1	1	1
17	Adjust for length of follow-up	1	1	1	1	1	1	1	0	1	1	1
18	Statistical tests appropriate	1	1	1	1	1	1	1	0	1	1	1
19	Compliance with intervention	1	1	1	0	1	1	1	1	1	1	1
20	Main measures valid and reliable	1	1	1	1	1	1	1	1	1	1	1
21	Recruited from the same population	1	1	1	1	1	1	1	1	1	1	0
22	Groups recruited same time	0	1	0	1	1	0	1	1	1	1	1
23	Randomized to intervention groups	0	1	0	1	1	0	1	0	1	1	0
24	Random assignment concealed	0	0	0	0	0	0	0	0	1	1	0
25	Adjustment for confounding	1	0	0	0	1	0	1	0	0	1	1
26	Loss to follow-up taken into account	1	1	1	0	1	1	1	0	1	1	0
27	Power estimation reported	0	1	1	1	1	0	1	1	0	1	1
	**SCORE**	**19**	**23**	**19**	**18**	**21**	**15**	**23**	**14**	**22**	**23**	**19**

## Appendix C CHEERS Checklist

**Table:**

CHEERS CHECKLIST	Beard	Bettger	Cottrel	Fatoye	Hollinghurst	Horton	Kloek	Mallet	Pastora	Tousignant	Zachweija
1	0	0	1	1	1	0	1	1	1	1	1
2	1	1	1	1	1	1	1	1	1	1	1
3	1	1	1	1	1	1	1	1	1	1	1
4	1	1	1	1	1	1	1	1	1	1	1
5	1	1	1	0	1	1	1	1	1	1	1
6	0	0	1	0	0	0	1	0	1	1	1
7	1	1	1	1	1	1	1	1	1	0	0
8	1	1	1	1	1	1	1	1	1	1	1
9	0	1	1	1	1	0	1	0	0	1	1
10	1	1	0	1	1	1	1	0	0	1	1
11	0	1	0	1	1	1	1	0	0	0	1
12	1	1	1	0	1	1	1	1	1	1	1
13	1	1	1	0	1	0	1	1	1	1	1
14	0	1	1	1	1	0	1	1	1	1	1
15	1	1	1	1	1	1	1	1	1	1	1
16	0	0	0	0	0	0	0	0	0	0	0
17	0	1	1	0	1	0	1	0	0	1	0
18	0	1	1	1	1	1	1	0	1	1	1
19	0	1	1	1	1	1	1	0	0	1	1
20	0	1	0	0	1	0	1	0	0	1	0
21	0	0	0	0	0	0	1	0	0	1	1
22	1	1	1	1	0	1	1	1	1	1	1
23	1	1	1	1	1	0	1	0	1	1	0
24	1	1	1	1	1	1	1	0	1	1	1
**SCORE**	**13**	**20**	**19**	**16**	**20**	**14**	**23**	**12**	**16**	**21**	**19**
